# Bioconversion of chitin waste through *Stenotrophomonas maltophilia* for production of chitin derivatives as a Seabass enrichment diet

**DOI:** 10.1038/s41598-022-08371-1

**Published:** 2022-03-21

**Authors:** Kumaran Subramanian, Deivasigamani Balaraman, Mani Panangal, Tentu Nageswara Rao, Elumalai Perumal, Amutha R, Alagappan Kumarappan, Pugazhvendan Sampath Renuga, Suresh Arumugam, Rajasekar Thirunavukkarasu, Wilson Aruni, Suliman Yousef AlOmar

**Affiliations:** 1 Centre for Drug Discovery and Development, Sathyabama Institute of Science and Technology, Chennai, Tamilnadu 600119 India; 2 Department of Biotechnology, School of Bio and Chemical Engineering, Sathyabama Institute of Science and Technology, Chennai, Tamilnadu India; 3 CAS in Marine Biology, Annamalai University, Cuddalore, Tamilnadu India; 4 Department of Biotechnology, Annai College of Arts & Science, Kumbakonam, Tamil Nadu India; 5 Department of Chemistry, Krishna University, Machilipatnam, Andra Pradesh India; 6 Department of Pharmacology, Saveetha Dental College and Hospital, Chennai, Tamil Nadu India; 7 Department of Biotechnology, Periyar University PG Extension Center, Dharmapuri, Tamil Nadu India; 8 Molecular Biology Laboratory (Pure Health), Al Qassimi Women’s and Children’s Hospital, Wasit Street, Sharjah, United Arab Emirates; 9 Department of Zoology, Annamalai University, Annamalai Nagar, Cuddalore, Tamilnadu 608002 India; 10 Department of Zoology, Arignar Anna Government Arts College, Thiruvalluvar University, Cheyyar, Tamilnadu 604407 India; 11 Central Research Laboratory, Meenakshi Medical College Hospital & Research Institute, Kanchipuram, Tamil Nadu India; 12 United States Department of Veterans Affairs, Loma Linda University, Loma Linda, CA 92354 USA; 13 Zoology Department, College of Science, Kind Saud University, Riyadh, 11451 Kingdom of Saudi Arabia

**Keywords:** Microbiology, Biotechnology, Industrial microbiology

## Abstract

Marine wastes pose a great threat to the ecosystem leading to severe environmental hazards and health issues particularly the shellfish wastes. The shellfish waste which contains half of the amount of chitin can be efficiently transformed into useful products. Various approaches for the hydrolysis of chitin like physical, chemical, and enzymatic processes are there. Still, the use of enzyme chitinase is well documented as an effective and eco-friendly method. The present study summarizes the isolation of chitinase enzyme producing bacteria from different shrimp waste disposal sites in Parangipettai (India), and the possible use of an enzyme hydrolyzate as an immunostimulant to Asian Seabass (*Lates calcarifer*)*.* The potential chitinase-producing bacteria were identified by 16S rRNA gene sequencing as *Stenotrophomonas maltophilia.* After purification, the chitinase specific activity was 5.01 (U/ml) and the protein content was 72 mg and the recovery rate was 48.06%. The optimum pH and temperature for the chitinolytic activity were 6.5 and at 35–50 °C, respectively. The animal experiment trial was done with our feed supplements which included 0.0 (control), 0.5%, 1% and 2% of chitin degraded product. All the supplementary feed had an optimal 42% (w/w) of crude protein. The feed protein level was 41–43% on average and gross energy was 13–17 kcal/g and the feed was observed to exhibit a significantly higher (p < 0.05) survival rate, condition factor, specific growth rates, and body weight gain was also found to be promising compared to other fishes fed with control diet only. The red blood cells (RBC) and white blood cell (WBC) counts were found to increase significantly after being challenged with infection in animals fed with chitin derivatives from 1st week to 3rd week when compared to the control. The hematocrit (Hct) values were low on the 2nd and 3rd week in infected fish fed with chitin derivatives. This low level was due to infection lyses of the red blood cells and increased nitro blue tetrazolium reduction. The control diet-fed fish showed 70% mortality but the chitin derivative supplemented fishes showed only 20% mortality post-infection. The results of the study encompass that the use of chitin-derivate enriched feed further is taken into large-scale approaches thereby benefitting the aquaculture sector.

## Introduction

Chitin is a structural polysaccharide, which is composed of a β-(1,4)-linked polymer of N-acetyl glucosamine (GlcNAc) is present in the fungal cell walls, arthropods exoskeleton, crustaceans, and nematodes outer shells^1^. The debris of a fish like the skin, head, tails, shells, scales, and backbones accumulate as a result of advanced seafood processing technology. Due to the lack of adequate management, these fish waste products after processing, which are of high-value goods remain fully untapped. Chitin has a broad range of applications in various industries such as food, biomedical and chemical industries^2^.

Chitinases are enzymes that can be used to manufacture chito-oligosaccharides. These enzymes have been found to show antibacterial agents, lysozyme inducer elicitors, and immune enhancers^3^. The prime role of this chitinase in the bioconversion of chitin into pharmacologically active products, N-acetylglucosamine, and chito-oligosaccharides^4^. Enzymes are preferred over chemical reactions for the eco-friendly production of chitin derivatives^5^. Antibiotic use in the aquaculture sector is becoming increasingly restricted around the world and there is no viable vaccine technique to combat infection and so making aquaculture scientists look for safe disease control strategies. There was a lot of use of probiotics, synbiotics, and immunostimulants, which are now replaced by prebiotics that are currently used by many farms. In this case, CS (chitosan) is a non-toxic natural substance and also an immunostimulant that boosts both humoral and cellular immune responses by enhancing phagocytosis in a variety of fish species, respiratory bursts, alternate complements, lysozyme activity, and antibacterial peptide activity in the blood.

It is also important to boost the non-specific defense mechanism in all vertebrates. Fishes live in a cool environment and have a short lifespan. This reduces the formation of specific immunity and hence the non-specific immunity is of prime importance for fishes to deal with pathogenic microorganisms^6^. Toward off the pathogens and induce resistance antibiotics, chemicals, and drugs are used^7^. There has been recent interest in the development and use of immunostimulants and immunomodulators that include levamisole, peptidoglycan, vitamins, β-glucan, chitin, chitosan yeast, plant and animal products for stimulating non-specific and humoral defense systems^8^. Immunostimulants are feed additives that can modulate the immunological response of fish and improve disease resistance. They can be generated from natural sources or synthesized. Therefore, people are starting to think about the immunological stimulants used in aquaculture.

The chitin derivative, N-Acetylglucosamine (GlcNAc) oligomers is proven to induce defense against pathogens in plants^9^. In addition, the oligomers also find their application as a sweetener and nutritional supplement. The industrial production of GlcNAc can be made by hydrolytic reaction using strong acid but generates waste products that have to be treated before disposal which is not cost-effective. So, the alternative for chemical treatment is the use of microbial chitinolytic enzymes for the productions of GlcNAc and chitin oligomers. The enzymatic process generates chitin derivatives in a cost-effective and eco-friendly manner. The present study focuses on the use of microbial chitinolytic enzymes for the generation of chitin derivatives and its use in enriching diet to enhance the innate immune response and disease resistance in cultured Asian Seabass (*Lates calcarifer*)*.*

## Materials and methods

### Preparation of colloidal chitin

A 5 g aliquot of chitin flakes was taken, mixed with 30 ml of 35.5% hydrochloric acid at 4 ºC, and incubated overnight. To this 250 ml of 50% chilled ethanol was added slowly with constant stirring at 4 °C. Colloids of chitin were observed in ethanol. This mixture was centrifuged at 10,000 rpm and the pellet was washed with distilled water till the pH reached 7.0^10^.

### Sample collection

The soil samples were collected from shrimp and crab waste disposable sites in Parangipettai (Lat. 11°29′ N, Long. 79°46′ E), Cuddalore District, Tamil Nadu, India. Parangipettai coastal environment is a public area very close proximity to the institute Centre of Advanced Study in Marine Biology. Since it is a public domain no special permission is required to access it and also for research activities. All the experimental study was carried out under the approved guidelines.

### Isolation and screening of chitinase producing bacteria

About 1 g of collected soil sample was diluted and plated in chitin minimal agar (colloidal chitin, 1%; KH_2_PO4, 0.3 g; K_2_HPO_4_, 0.3 g; NaCl, 4 g; MgSO_4_·7H_2_O, 0.5 g; Agar, 1.5 g per liter). The inoculated plates were incubated at 28 °C for 72 h. After incubation, bacterial isolates which produced a zone of clearance around the colony were selected as the chitinase-producing bacteria for further studies^11,13^.

### Culture condition and production

A chitin minimal medium (CMM) containing 1% (w/v) colloidal chitin, 0.7% (w/v) K_2_HPO_4_; 4% (w/v) KH_2_PO_4_; 0.3% (w/v) NaCl; 0.5% (w/v) MgSO_4_·7H_2_O; and 0.5% (w/v) peptone, pH 7.2 was prepared for chitinase production and 50 ml of medium was dispensed in 250 ml of Erlenmeyer flasks and sterilized at 110 °C for 15 min. Each flask was inoculated with 1 ml aliquot of 18 h old seed culture and incubated at 37 °C at 100 rpm for 5 days. Culture broth has been centrifuged for 10 min at 8000 rpm (Sigma laboratory centrifuge 4K15), and the supernatant was used as a crude enzyme^12^.

### Chitinase assay

About 2.5 ml aliquot of phosphate buffer saline was added in 1% of colloidal chitin (substrate) followed by preparation of crude enzyme of 0.5 ml. The tubes were incubated at 45 °C for 1 h. After incubation, the reaction was stopped by adding 3 ml of 10% dinitro salicylic acid (DNSA) followed by heating in boiling bathwater. The colored solution was then centrifuged for 5 min at 8000*g* and the resulting supernatant was measured in a spectrophotometer at 540 nm wavelength. The reduction of sugar has been derived from a standard glucose curve. One unit of enzyme is known as the enzyme quantity that catalyzes the release of 1 μM of reduced sugar per minute under assay^13^.

### Characterization of isolates

Chitin degrading isolates were characterized by studying their morphological and biochemical characteristics. The isolate results were compared with Bergey’s manual of determinative bacteriology. Further, a potential chitinase-producing bacterial strain was characterized by 16 S rRNA gene sequencing.

### Molecular characterization

The phenol–chloroform technique was used to extract genomic DNA from the bacterial isolates. The agarose gel (0.8%) was used to analyze the isolated DNA and NanoDrop 1000 (Thermo Fisher Scientific, Wilmington, DE, USA) was used to quantify it. PCR was used to amplify the bacterial strains 16S rRNA gene sequence using universal bacterial primers 27F and 1492R, as well as GeNeiTM PCR Master Mix (Genei, Bangalore, India). For the PCR protocol^13^, a thermal cycler (Genei, Bangalore, India) was used: 94 °C for 5 min (initial denaturation), 94 °C for 1 min (denaturation), 53 °C for 30 s (annealing), 72 °C for 90 s (elongation)—35 cycles and 72 °C for 7 min (final extension).

### Phylogenetic analysis

The 16S rRNA gene sequences were amplified by polymerase chain reaction (PCR) using universal primers 27F and 1292R. The sequences of the amplified 16S rRNA genes CHI2 were obtained by an automated sequencer (Bioserve, Hyderabad) almost in full length. Clustal X mega program edited the sequences, and the National Center for Biotechnology Information (NCBI) database conducted a BLAST scan to find the nearest sequence. The sequencing findings were used to perform homology searches and the neighbor-joining method a phylogenetic tree was developed. The sequences of partially 16S rRNA gene sequences were deposited in the GenBank database, and it was given an accession number.

### Extraction and purification of chitinase

At 4 °C, the crude enzyme was precipitated with varied doses of ammonium sulfate, ranging from 50 to 80% (w/v). The solution was slowly stirred and kept at 4 °C for 30 min to dissolve all concentrations of the ammonium sulfate. The precipitate was centrifuged at 10,000 rpm for 30 min at 4 °C; the precipitated protein was recovered and mixed with Tris–HCl buffer pH 7.5. The activity of chitinase and the amount of protein in the samples were examined. After precipitation, dialysis was employed to remove excess salt, organic solvent, and low molecular weight inhibitors. The protein sample was dialyzed overnight at 4 °C with 2–3 times buffer changes against 20 mM Tris HCl buffer at pH 7.5.

The dialyzed enzyme was put to a Sephadex 75 column (20 cm × 1.5 cm) and equilibrated with 20 mM Tris HCl buffer (pH 8.0). The enzyme was eluted with 20 mM Tris HCl and a flow rate of 1 ml/5 min was maintained. A 280 nm absorbance scale was used to determine the protein content. Peaks demonstrating enzyme activity were combined and identified as distilled enzymes. The effects of temperature and pH on the function and stability of the enzymes were investigated using this diluted enzyme solution.

### Determination of optimal temperature and pH for enzyme activity

The reaction solution was incubated at different temperatures to determine the optimum temperature for enzyme activity. The thermostability of the enzyme was tested by incubating 0.2 ml of pure enzyme in 50 mM Tris–HCl (pH 9.0) for 1 h at various temperatures, including 30, 35, 40, 45, 50, 55, and 60 °C. To test the effect of pH on enzyme activity, researchers used five different buffer systems (all 50 mM): citric acid-Na_2_HPO_4_ (pH 5 to 6), phosphate buffer (pH 6 to 8), boric acid-NaOH buffer (pH 8 to 9.5), phosphate-NaOH buffer (pH 9.5 to 12), phosphate-NaOH buffer (pH 9.5 to 12), phosphate-NaOH buffer (pH 12–13 The stability of the enzyme was tested for 1 h by adding enzyme solution at various pH levels, and the activity was measured.

### Experimental trial

#### Acclimatization of experimental fishes

Healthy Asian Seabass (*Lates calcarifer*) (20–30 g in weight) were procured from grow-out ponds of RGCA (Rajiv Gandhi Centre for Aquaculture), Karaikal, Tamil Nadu, India. The fishes (n = 300) were transferred to a 500-L fiber tank filled with UV treated seawater and continuous aeration was provided. Fishes were acclimated to these conditions for at least 7 days before initiating the experiment and fed with an artificial pellet two times a day. The experiment was carried out with the optimal water temperature of 27.2 °C ± 0.6, the salinity of 27 ± 2.2 ppt, the dissolved oxygen concentration of 5.6 ± 0.5 (mg/l), and pH 8.0 ± 0.3 respectively. The ammonia (0.01, 0.02, 0.03 and 0.04 mg/ml) and nitrite (0.01, 0.05, 0.05 and 0.06 mg/ml) contents in the water were maintained at low level. This study was carried out in compliance with the CPCSEA safety guidelines and following ARRIVE guidelines (https://arriveguidelines.org) for the reporting of animal experiments. The study was approved by the Institutional Animal Ethics Committee (IAEC) 1793/PO/ReBi/S/2014/CPCSEA and by the Centre for Lab animal science, Sathybama Institute of Science and Technology.

#### Chitin degraded product enriched diet

Based on the chitinase optimization, the chitin degraded product was obtained. The degraded product was lyophilized and dispensed in a little volume of Milli-Q water. To prepare the experimental diet was mixed evenly to basal diet and air-dried aseptically. The control was sprayed with Milli-Q water. The prepared pellets were dried using an oven at 35 °C for 12 h and stored at − 20 °C for further use.

As indicated in Table 1, five dietary supplements including 0.0 (control), 0.5%, 1% , and 2% chitin degradation product feed were developed to give optimal 42% (w/w) crude protein. In addition, different quantities of the chitin derivatives feed supplement was mixed in 100 mL/kg feed and homogenized for 30 min with the other components to make fine consistency dough (Table 1). The dough was mixed in a mixer at the same time and pelleted using a paste extruder with a diameter of around 1 mm. The feed pellets were dried for 24 h at 55 °C and stored at − 20 °C for future use.

**Table 1:** Chemical composition of feed supplements (% dry weight) containing various levels of chitin derivatives.

Components	Chitin (g/kg diet)			
	0.0 (Control)	0.5	1.0	2.0
Fish meal	134	134	134	134
Soya bean meal	300	300	300	300
Chicken meal	120	120	120	120
Groundnut cake	233	233	233	233
Wheat bran	100	104.5	105.5	104
Codfish oil	28	23	23	24
Corn oil	10	10	10	10
Vitamins	15	15	15	15
Minerals	25	25	25	25
Starch	35	35	35	35
Chitin derivatives	0.0	0.5	1	2.0
Total	1000	1000	1000	1000
**Proximate chemical analysis (%)**				
Dry matter	10.2	10.1	10.2	10.3
Crude protein	42.36	42.56	42.52	42.65
Ether extract	6.8	6.7	6.9	6.8
Total ash	5.1	5.3	5.2	5.4
Crude fiber	6.1	6.4	6.2	7.2
Nitrogen free extract^a^	33.2	32.1	33.5	33.2
Gross energy (Kcal/100 g)^b^	14.2	15.2	15.1	15.2

^a^Nitrogen free extract (NFE) = 100 – (protein % + lipid % + total ash % + crude fiber %).

^b^Gross energy was calculated according to NRC (1993) as 5.65, 9.45, and 4.11 kcal/g for protein, lipid, and carbohydrates, respectively.

### Experimental design

Four rectangular fiberglass tanks (100 L) were placed with continuous aeration. The fishes (20 ± 4 g) were randomly distributed in four experimental groups with triplicate. One tank was kept as control (T1), one with 0.5% chitin degraded product (CDP) (T2), 1% CDP (T3), and 2% CDP (T4), The fish were fed the basal feed during the acclimation period. The fish in the experiment were fed twice a day. After that, each tank was assigned to one of three duplicates of four different experimental meals at random. For 45 days, three groups of fish were fed one of the experimental diets twice daily (10:00 and 17:00 h) at about 4% of wet body weight per day at the start and 5% of wet body weight per day at the end of the feeding study. On the 14th day, fishes from the T3 and T4 group were inoculated intraperitoneally with 100 μl of *Aeromonas hydrophilia* (MTCC) suspension. Randomly 3 fishes were collected from each tank on the 1st, 2nd, and 3rd week of post-infection and anesthetized using MS-222.

### Growth

#### Feed consumption measuring and growth rate

The fish fed with CDP/kg feed from each tank were taken, measured, and documented after the feeding trial analysis was completed on the 45th day. Enumerating the individuals in each tank was also used to calculate the fishes survival rate. Weight gain (WG), specific growth rate (SGR), feed intake (FI), feed conversion ratio (FCR), and protein efficiency ratio (PER) was used to calculate the efficiency of fish productivity and diet consumption using equations.

$${\text{SGR}} = {1}00 \, \times \, \left[ {{\text{Ln final weight}} - {\text{Ln initial weight}}} \right]{\text{/total duration of the experiment}}.$$

$${\text{FCR}} = {\text{Feed given }}\left( {\text{dry weight}} \right)/{\text{weight gain }}({\text{wet weight}}).$$

$${\text{Survival }}\left( \% \right)=\left( {{\text{final number of fish}}/{\text{initial number of fish}}} \right)\, \times \,{1}00.$$

$${\text{Feed conversion ratio }}\left( {{\text{FCR}}} \right) = {\text{Feed consumption }}\left( {\text{g}} \right)/{\text{Weight gain }}({\text{g}}).$$

$${\text{Protein efficiency ratio }}\left( {{\text{PER}}} \right) = {\text{Total wet weight gain }}\left( {\text{g}} \right)/{\text{Crude protein fed }}({\text{g}}).$$

### Non-specific immune response in Asian Seabass

#### RBC and WBC count

Haemocytometer was used to evaluate the number of RBC and WBC^14^. The number of cells per ml of the blood sample was determined using the formula.

$${\text{Number of cells}} = \left( {{\text{Number of cells counted }} \times {\text{ dilution}}} \right) \, \left( {{\text{ml}}^{{ - {1}}} } \right)/\left( {{\text{Area counted }} \times {\text{ depth of fluid}}} \right).$$

#### Hematocrit p-value

The percentage of blood cells in the total volume of blood was determined as described by Goldenfarb et al.^15^.

#### Serum bactericidal activity

For evaluating serum anti-bactericidal activity, *A. hydrophila* was mixed with serum and incubated for 1 h at 37 °C. Then, 0.1 ml of the suspension was placed on nutrient agar incubated at 24 h at 37 °C. After incubation, the number of viable bacteria was calculated^15^.

#### Respiratory burst and lysozyme activity

The formation of oxidative radicals from neutrophils in blood during respiratory burst was evaluated by NBT assay, as described by Anderson and Siwick^16^. NBT assays were modified and used to quantify the formation of oxidative radicals by neutrophils in blood during the respiratory burst, In blood and 0.2% NBT were mixed in equal parts (1:1), incubated at room temperature for 30 min, and then 50 L were extracted and dispensed into Eppendorf tubes. 1 mL dimethylformamide (Sigma, USA) was used to solubilize the reduced formazan product, which was centrifuged for 5 min at 2000 rpm. Finally, using a micro reader and a supernatant, the extent of reduced NBT was evaluated at an optical density of 540 nm.

#### Lysozyme activity

Lysozyme activity was determined by using the turbidimetric method according to Abu-Elala et al.^17^. In a 96 well plate with 50 µl PBS, pH 5.8, 50 μl of serum were put in triplicate. The serum was serially diluted till the last well after mixing. Finally, in the last well, 50 µl of the sample was eliminated. *Micrococcus lutius* (75 mgml1in phosphate buffer) was introduced to each well in a volume of 125 µl. In an ELISA reader, the drop in absorbance at 450 nm was measured from 0 to 15 min at room temperature. Using hen egg-white lysozyme (Sigma, USA) as a standard, the lysozyme activity was converted to lysozyme concentration.

#### Disease resistance and survival experiment

The disease-resistant experiment was conducted following the methodology of Harikrishnan et al.^18^ with slight modifications. The fishes from the experimental and control group were inoculated with *Aeromonas hydrophilia* (1 × 10^8^ cells ml^−1^). The fishes were observed for mortality and clinical symptoms.

### Statistical analysis

All of the analyses were done in triplicate, and the results were expressed as mean standard error. Using SPSS software (version 17.0, SPSS Inc., USA), all of the collected data were subjected to one-way ANOVA analysis. ANOVA was performed to find differences between groups, and Duncan’s multiple range tests were employed to evaluate the difference in means.

### Ethical approval

The present study follows institutional guidelines mandatory for human and animal treatment and complies with relevant legislation ethical approval from the institute for conducting the research. The study was approved by the Institutional Animal Ethics Committee (IAEC) 1793/PO/ReBi/S/2014/CPCSEA and by the Centre for Lab animal science, Sathybama Institute of Science and Technology.

## Results and discussion

### Isolation of chitin producing strains

In the present study chitin degrading bacteria were isolated from the site of shrimp waste disposable sites of Parangipettai (Lat. 11°29′ N, Long. 79°46′ E), Cuddalore district. Screening of chitin degrading activity was carried out on chitin minimal agar plates, using colloidal chitin as the indicator. Among the isolates, 14 bacterial isolates with the zone of inhibition study were selected for further studies. The marine milieu naturally contains chitinolytic bacteria that play a major role in the degradation of chitin^19^. All strains were further screened in chitin minimum medium and incubated for 5 days following incubation with two (CHI2) bacterial isolates that demonstrated the highest chitinase activity. The selected strains were able to degrade chitin under aerobic growth conditions by using the colloidal chitin as the carbon source. This was visibly confirmed by the presence of a zone of clearance around it. In our earlier study, we isolated and characterized an *Achromobacter xylosoxidans* bacterium from the shrimp disposal site.

In the present study, a total of 14 different strains of marine bacteria with chitin degrading potential were isolated. Out of that, the bacterial strain which had high chitinase activity was taken for further analysis. We found that the *Achromobacter xylosoxidans* strain from the previous work and the *Stenotrophomonas maltophilia* isolates from the present study were able to produce chitinase, which in turn can degrade chitin along with the concomitant production of N-acetyl-d-glucosamine^13^.

For accessing the degradation efficiency, 18 h broth culture was inoculated on chitin minimal broth and incubated under optimal conditions. After 5 days of incubation in a shaker incubator, the culture broth was centrifuged at 8000 rpm for 4 °C and the supernatant was collected for chitinase activity calculation. After 5 days of incubation, the bacteria showed major degradation of chitin.

### Screening of chitinase producing bacteria

Chitin degrading ability or chitinase activity of culture supernatants was estimated. The bacteria CHI2 showed maximum chitinase activity of 1.6 U/ml. The chitin might get hydrolyzed to chitobiose and N-acetylglucosamine. The potent isolate utilizes chitin as the sole carbon source and possesses maximum chitinase activity, which was selected for further studies.

### Identification of potent bacterial isolates

The potent CHI2 isolate was identified using morphological and molecular analyses. The CHI2 is a Gram-Negative, motile, catalase-positive, citrate-positive, oxidase-negative, glucose-negative bacterium. Further, these strains were identified through the genetic marker and molecular identification.

**Figure 1:**
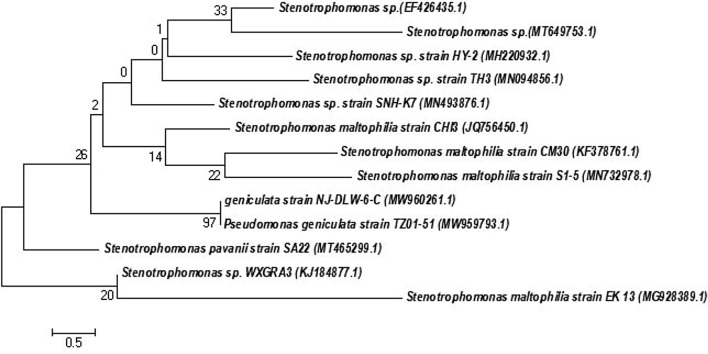
Phylogenetic tree of potent bacterial strain *Stenotrophomonas maltophilia*.

The 16S rRNA gene sequence showed high similarities to those of genera consisting of *Stenotrophomonas maltophilia.* The partial 16S rRNA gene sequence of *Stenotrophomonas maltophilia* was deposited in the NCBI database under the accession number JQ756450. The neighbor-joining method was used for the construction of a phylogenetic tree for the selected strains (Fig. 1). Puspita et al.^20^ isolated a high level of enzyme-producing bacteria including *Stenotrophomonas maltophilia*.

### Purification of chitinases

The purification step was started with the ammonium sulfate precipitation technique. Screening of ammonium sulfate from 50 to 80% (w/v) concentration was done and resulted that 75% (w/v) of ammonium sulfate was the optimum concentration to precipitate chitinase out. The chitinase fraction was obtained as a part of the fourth protein peak. The chitinase production was known to be enhanced by-products (N-acetylglucosamine, glucosamine, and chitobiose) of chitin hydrolysis^21^.

For *S. maltophilia*, the chitinase specific activity was 5.01 and the protein content was 72 mg and the recovery was 48.06%. Thus, the chitinase was purified 5.7 fold by the three steps and the overall yield was 27%. The bacteria CH112 produce 1.6 U/ml of chitinase at 5 days. The chitinase act on chitin and convert it to chitobiose and N-acetylglucosamine. Sashiwa et al.^22^ reported the synthesis of chitinase enzyme using *Aeromonas hydrophila* H-2330 for the production of GlcNAc.

### Effect of pH, the temperature on the stability and activity of chitinase

**Figure 2:**
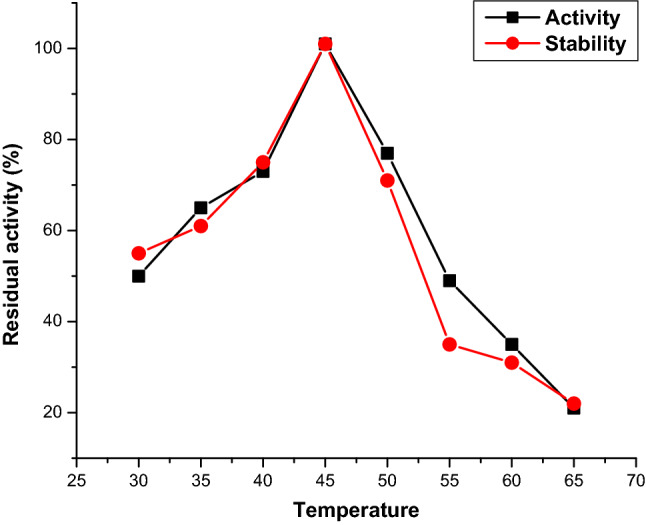
Effect of temperature on activity and stability of purified chitinase from *S. maltophilia*.

It was found after 48 h of inoculation *S. maltophilia* was detected with chitinolytic activity and it showed maximum growth and enzyme activity after 168 h. The optimal temperature for the activity of chitinase from *S. maltophilia* was at 45 °C. The stability of the enzyme was 95% at 45 ºC. The enzyme was active in a broad temperature range (35–50 °C). Minor variations in temperature intensity have not led to a significant reduction in enzyme activity**.** There was no enzyme activity when incubated above 65 °C (Fig. 2).

**Figure 3:**
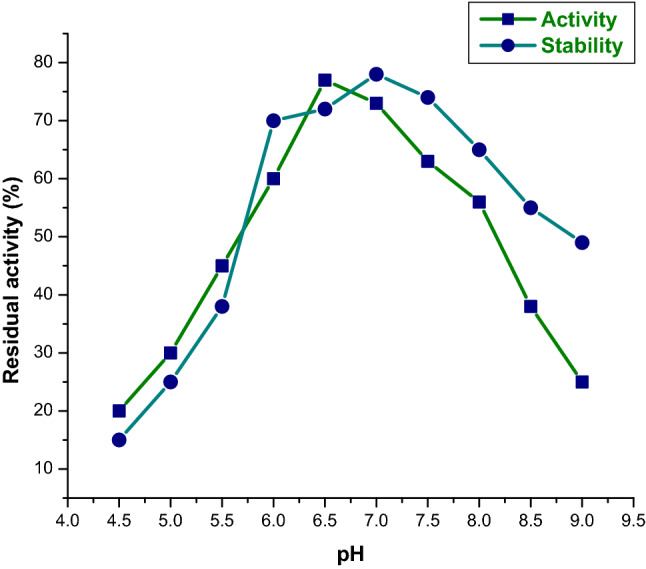
Effect of pH on activity and stability of purified chitinase from *S. maltophilia*.

The chitinase synthesized from *S. maltophilia* was active in large ranges of pH from 5 to 7 and maximum at 6.5 pH. Chitinase activity decreased at pH above and below 6 because of differences in the protein molecules with ionic residues. The enzyme’s active site contains an amino acid that plays a major role in the enzyme conformation. Jankiewicz et al.^23^, reported that the pH optimum for colloidal chitin hydrolysis by the enzyme studied was 6.8. The observed enzyme only maintained 10 percent of its average activity at pH 5.5 and 8.5. According to previous reports, the enzyme might denature after prolonged incubation at unsuitable pH conditions. The result is similar to previous research that chitinolytic enzyme stable at pH range from 4.0 to 8.0 (Fig. 3).

Chitinase isolated from *Stenotrophomonas maltophilia* was active between wide ranges of pH between 5 and 7 and optimum being at 6.5 pH. It is 78% stable at pH 7 and 77% active at pH 6.5. Zarei^24^ has also found the use of *Serratia marcescens* for optimal chitinase production in pH. Similar results with *Streptomyces* sp. were reported by Narayana and Vijayalakshmi^25^.

### Growth

Fishes fed with a basal diet enriched with chitin derivatives showed (Table 2) significantly higher (p < 0.05) survival rate, condition factor, specific growth rates, and body weight gain as compared to fishes fed with basal diet only. The feed conversion ratio (FCR) was also increased on feeding with chitin derivatives. The diet supplemented with vitamin C enhances specific growth rates up to 1000 mg kg^−1^ in Indian major carp, rohu (*Labeo rohita*)^26^.

**Table 2:** Growth and feed consumption efficiency of *Lates calcarifer* supplemented with varying quantities of chitin derivatives product (CDP).

Parameters	Chitin derivative loaded with feed (g/kg diet)				P-value
	0.0 (Control)	0.5	1.0	2.0	
Initial weight (g)	20.30 ± 0.22^a^	20.63 ± 0.12^a^	20.56 ± 0.09^a^	20.53 ± 0.26^a^	> 0.452
Final weight (g)	35.33 ± 0.43^a^	33.73 ± 0.44^a^	40.80 ± 0.20^b^	36.3 ± 0.56^a^	< 0.001
Weight gain (g)	15.44 ± 0.36^a^	13.53 ± 0.93^a^	19.71 ± 0.96^b^	13.89 ± 1.93^a^	< 0.001
Weight gain (%)	75.95 ± 1.68^a^	65.98 ± 4.53^a^	96.39 ± 4.96^b^	68.02 ± 9.11^a^	< 0.001
Specific growth rate (SGR) (%)	3.11 ± 0.01^b^	3.19 ± 0.03^c^	3.01 ± 0.03^a^	3.17 ± 0.05^b,c^	< 0.001
Total food intake (TFI) (g)	45.75 ± 0.34^a^	46.15 ± 0.26^a^	46.02 ± 0.31^a^	45.93 ± 0.26^a^	> 0.458
Feed conversion ratio (FCR) (g/g)	1.76 ± 0.03^b^	2.12 ± 0.10^c^	1.51 ± 0.10^a^	1.20 ± 0.23^b,c^	< 0.002
Protein efficiency ratio (PER) (g/g)	0.84 ± 0.02^a^	0.73 ± 0.10^a^	1.01 ± 0.10^b^	0.76 ± 0.10^a^	< 0.001
Survival rate (%)	100	100	100	100	–

Values within the same row having different superscripts are significantly different (P < 0.05). Data were presented as mean ± SE (n = 3). ^a,b,c^ Superscripts represents significant variation based on DMT (Duncan multiple test range).

### Immunological parameter

**Figure 4:**
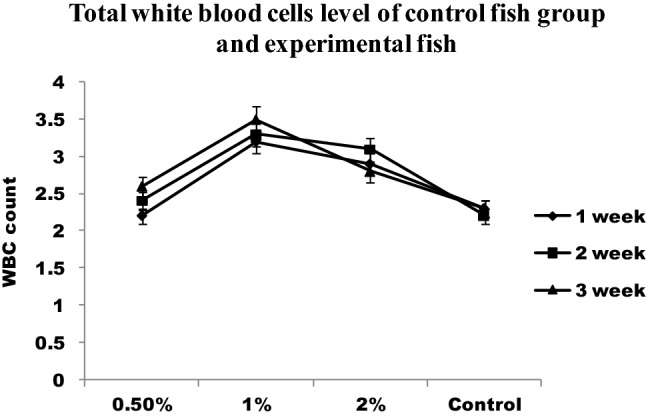
Total white blood cells level of control fish group and experimental fish fed CDP with basal diet.

**Figure 5:**
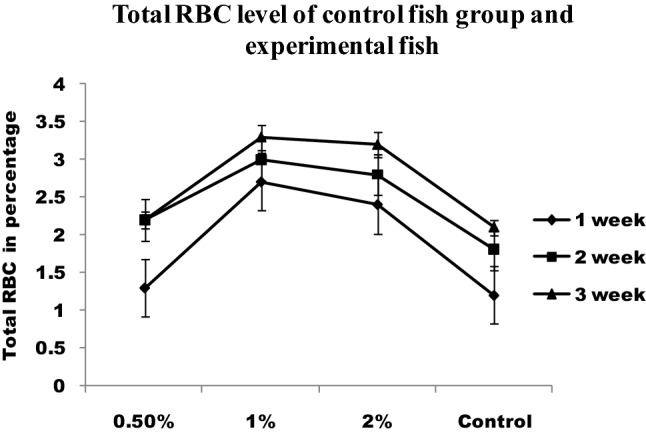
Total RBC level of control fish group and experimental fish fed with CDP with basal diet.

**Figure 6:**
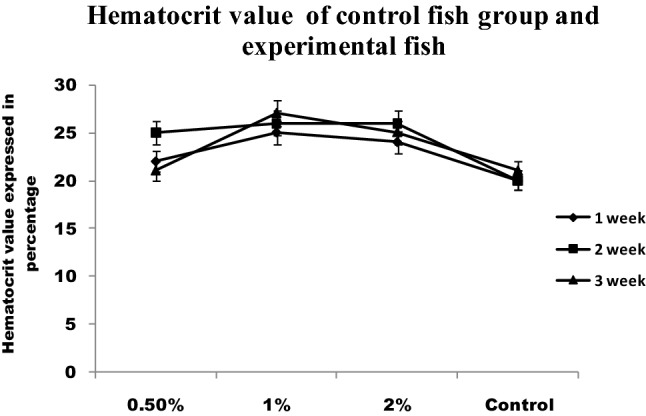
Hematocrit value of control fish group and experimental fish fed with CDP with basal diet.

RBC and WBC increased significantly in infected fish fed with chitin derivatives from 1st week to 3rd week when compared to control (Figs. 4 and 5). The red blood cells level decreased in infected fish without chitin derivative feed. During the first week, the RBC level is significantly higher followed by pathogenic infections in fishes. The hematocrit value (Hct) significantly decreased in infected fish (Fig. 6). The control fishes have normal value but the chitin degraded product supplementation feed fishes have significantly higher than the control fishes. The chitin derivatives influenced infected fish hematocrit (Hct) values to be low on the 2nd and 3rd week due to infection lysis the RBCs. In a study, the Ht had significantly increased in fish fed with chitin derivatives diet against pathogen^27^.

Fish-fed diets with chitin derivatives had the highest total. The CDP diet with infected fishes has more counts compared to control and without chitin derivatives with infected fishes have significantly reduced on 1st week to 3rd week. The serum bactericidal activity shows the CDP diet enhanced the blood system because the Asian Seabass serum after being fed with the CDP diet shows more activity against pathogenic *Aeromonas hydrophilia* infection.

**Figure 7:**
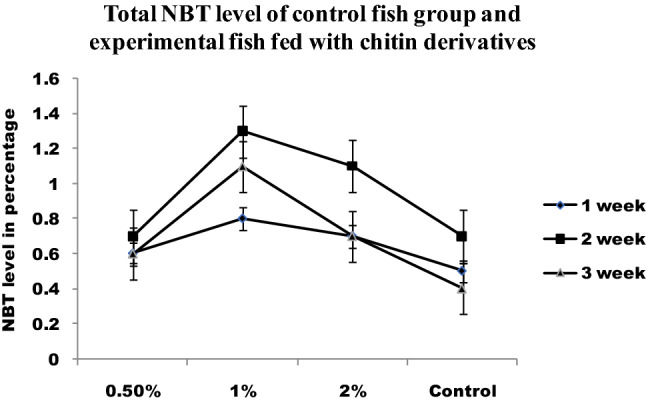
Total NBT level of control fish group and experimental fish fed with chitin derivatives.

**Figure 8:**
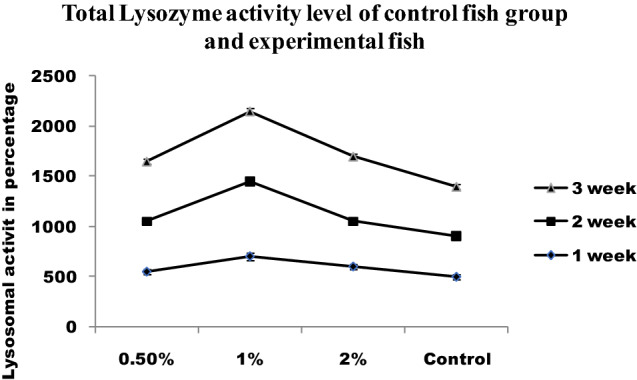
Total Lysozyme activity level of control fish group and experimental fish fed CDP with basal diet.

The increased NBT reduction reveals the influence of dietary supplements on neutrophil respiratory burst (Fig. 7). Infected fishes fed with a normal diet had increased respiratory burst activity as compared to control. The chitin derivatives supplementation enhanced respiratory burst activity. On the other hand, chitin derivatives with infection also proved increased respiratory burst activity. The chitin derivatives supplemented feed showed increased lysozyme activity in fishes after the first week (Fig. 8).

According to statistics, contemporary growing fish producers pay around lakhs (INR) in importation feed costs for a single pond. This low-cost feed product does not doubt reach aqua farmers and will take the lead in the global and Indian aquaculture feed markets. Several authors proved chitin enriched fed diet of the fish improved the immune system. Even though chitin and chitosan were found to possess biological activity it has been less exploited in the food and biomedical industry since it has low solubility. Instead of chitosan, chitosan oligosaccharides (COS) obtained from chitosan hydrolysis are easily soluble in water because of shorter chain length and the presence of free amino groups in d-glucosamine units^28^.

### Disease resistance

**Figure 9:**
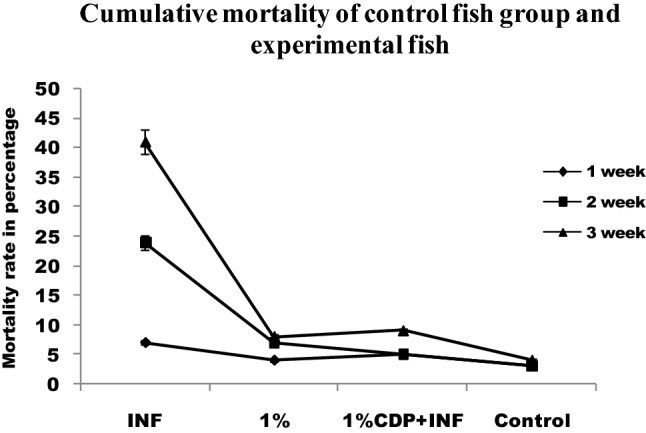
Cumulative mortality of control fish group and experimental fish fed with CDP with basal diet.

The control diet-fed fish showed 70% mortality upon infection but chitin derivative supplemented diet fishes showed only 20% mortality after infection (Fig. 9). The present study demonstrated that the development of fish feed using chitin derivatives produce a significant effect on the non-specific immunity and disease resistance in fish.

## Conclusion

Aquaculture industries mainly rely on antibiotics, chemicals, and vaccines to overcome pathogenic infection and disease prevention. However, at present, the excessive and strong dosed use of modern antibiotics makes the pathogens resistant even to strong drugs and this has made to prohibit the usage of antibiotics in aquaculture practice today. The present experimental study strategizes to use feed as immunomodulators and disease resistance by using a lab-made biological feed through a bioremediation approach. With the results observed we see the application of chitin and its derivatives as an immunostimulant for the growth of farm-reared Asian Seabass (*Lates calcarifer*). It was also found to be an effective immune booster as well as a natural feed. This type of natural feed tends to be a promising approach in bioremediation. Chitin as a feed showed zero adverse effect in *Lates calcarifer*, which depicts it as an organic natural feed for wide use in the aquaculture industry.
